# Two types of highly ordered micro- and macrochromosome arrangement in metaphase plates of butterflies (Lepidoptera)

**DOI:** 10.3897/CompCytogen.v13i1.32614

**Published:** 2019-01-14

**Authors:** Vladimir A. Lukhtanov

**Affiliations:** 1 Department of Karyosystematics, Zoological Institute of the Russian Academy of Sciences, Universitetskaya nab. 1, St. Petersburg 199034, Russia Zoological Institute of the Russian Academy of Sciences St. Petersburg Russia; 2 Department of Entomology, St. Petersburg State University, Universitetskaya nab. 7/9, St. Petersburg 199034, Russia St. Petersburg State University St. Petersburg Russia

**Keywords:** Asymmetrical karyotype, DNA barcoding, bivalent, COI, holocentric, holokinetic, kinetochore, meiosis, metaphase, spindle, spermatocyte, Lepidoptera, Nymphalidae, Danainae, Ithomiini, Peru

## Abstract

In karyotype of many organisms, chromosomes form two distinct size groups: macrochromosomes and microchromosomes. During cell divisions, the position of the macro- and microchromosomes is often ordered within metaphase plate. In many reptiles, amphibians, birds, insects of the orthopteran family Tettigoniidae and in some plants, a so called “reptilian” type organization is found, with microchromosomes situated in the center of metaphase plate and with macrochromosomes situated at the periphery. An opposite, “lepidopteran” type is known in butterflies and moths (i.e. in the order Lepidoptera) and is characterized by macrochromosomes situated in the center and by microchromosomes situated at the periphery. The anomalous arrangement found in Lepidoptera was previously explained by holocentric organization of their chromosomes. Here I analyse the structure of meiotic metaphase I plates in ithomiine butterfly, *Forbestraolivencia* (H. Bates, 1862) (Nymphalidae, Danainae, Ithomiini) which has a clear “reptilian” organization, contrary to previous observations in Lepidoptera. In this species large bivalents (i.e. macrochromosomes) form a regular peripheral circle, whereas the minute bivalents (i.e. microchromosomes) occupy the center of this circle. The reasons and possible mechanisms resulting in two drastically different spatial chromosome organization in butterflies are discussed.

## Introduction

The spatial organization of chromosomes and chromosome bivalents may be highly ordered during interphase and cell divisions (White 1973, Cremer et al. 1982, 2017, Solé et al. 2017, Sarrate et al. 2018). For example, a special (“reptilian” according to White 1973) type of the ordered metaphase plate organization was found in taxa with asymmetrical karyotype including groups of micro- and macrochromosomes, e. g. in many reptiles, amphibians, birds, in some insects and in some plants (White 1973, Lewitsky 1976). In these taxa, the microchromosomes occupy position in the center of metaphase rosette inside of the spindle, and the macrochromosomes are situated at the periphery and form a ring around the spindle.

In our previous work we demonstrated that butterflies and moths have inverted spatial karyotype organization at the first male meiotic metaphase, with larger chromosomes situated in the center and smaller chromosomes situated at the periphery (Lukhtanov and Dantchenko 2002). The latter observation has been confirmed in numerous subsequent studies (e.g. Vershinina and Lukhtanov 2010, Przybyłowicz et al. 2014, Vershinina et al. 2015, Lukhtanov 2015).

After our research was published (Lukhtanov and Dantchenko 2002), a study appeared focused on the chromosome evolution in Neotropical Danainae and Ithomiinae (Lepidoptera, Nymphalidae) (Brown et al. 2004). Although the spatial organization of chromosomes was out of the focus of this study and was not discussed at all, the article provided numerous microphotographs that demonstrated the central position of larger bivalents at the male first meiotic metaphase, but also a single figure (fig. 23, *Forbestraproceris* Weymer, 1883) in which this order was inverted. Therefore, during the expedition of St. Petersburg University to Peru in 2013, I paid special attention to collecting representatives of the genus *Forbestra* R. Fox, 1967 as well as other taxa of the tribe Ithomiini. Description of karyotypes and bivalent spatial organization in three species of the Ithomiini is given below.

## Material and methods

### Samples

**Figure 1.**
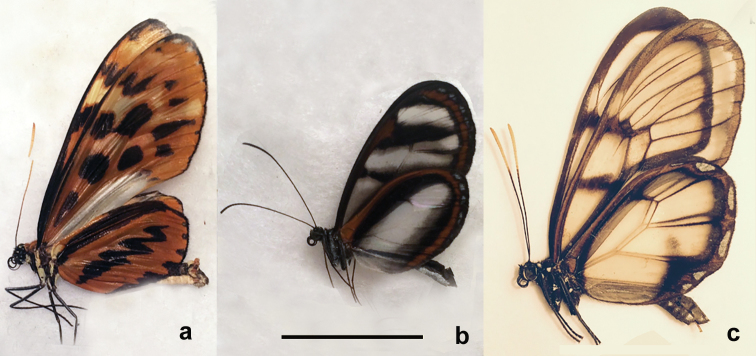
The analyzed samples of Ithomiini**a***Forbestraolivenciaolivencia* (Bates, 1862) (form huallaga Staudinger, [1884]), NOB003-17 (CCDB-23376_A03, 2013_A109), Peru, 50 km S of Ikitos, 04°11'47"S; 73°28'39"W, 114 m, 30 August 2013, V. Lukhtanov leg **b***Oleriagunillaserdolis* (Haensch, 1909), NOB012-17 (CCDB-23376_A102, 2013_A127), Peru, Tingo Maria, 09°21'02"S; 76°03'21"W, 835 m, 3 September 2013, V.Lukhtanov leg **c***Godyrisdircenna* (C. Felder & R. Felder, 1865), NOB026-17 (CCDB-23376 C02, 2013_A145), 09°29'43"S; 75°58'01"W, 800 m, 6 September 2013, V.Lukhtanov leg. Scale bar: 20 mm in all figures.

Karyotypes were studied in two specimens of *Forbestraolivenciaolivencia* (H. Bates, 1862) (form huallaga Staudinger, [1884]), four specimens of *Oleriagunillaserdolis* (Haensch, 1909) and two specimens of *Godyrisdircenna* (C. Felder & R. Felder, 1865). The information on localities where the specimens were collected is presented in caption to the Figure 1. The samples were identified through their comparison with the butterflies figured at Butterflies of America site (https://www.butterfliesofamerica.com/L/Nymphalidae.htm). The specimens are deposited in the Zoological Institute of the Russian Academy of Sciences, St. Petersburg, Russia.

Standard *COI* barcodes (658-bp 5’ segment of mitochondrial cytochrome oxidase subunit I) were obtained for the sample NOB003-17 (CCDB-23376_A03, 2013_A109) of *F.olivencia*, for the samples NOB008-17 (CCDB-23376_A08, 2013_A121), NOB009-17 (CCDB-23376_A09, 2013_A122), NOB010-17 (CCDB-23376_A10, 2013_A123) and NOB012-17 (CCDB-23376_A102, 2013_A127) of *O.gunilla* and for the sample NOB026-17 (CCDB-23376 C02, 2013_A145) of *G.dircenna*. The barcodes were obtained at the Canadian Centre for DNA Barcoding (CCDB, Biodiversity Institute of Ontario, University of Guelph) using standard protocols (Hajibabaei et al. 2005, Ivanova et al. 2006 and deWaard et al. 2008). These DNA barcodes were used to confirm the species identification (http://boldsystems.org/index.php/IDS_OpenIdEngine).

### Chromosomal analysis

Gonads were removed from the abdomen and placed into freshly prepared fixative (3:1; 96% ethanol and glacial acetic acid) directly after capturing the butterfly in the field. Testes were stored in the fixative for 3–36 months at +4 °C. Then the gonads were stained in 2% acetic orcein for 30–60 days at +18–20 °C. Spatial organization of meiotic bivalents was studied in intact (not squashed) spermatocytes using protocol described in Vishnevskaya et al. (2016).

## Results and discussion

**Figure 2.**
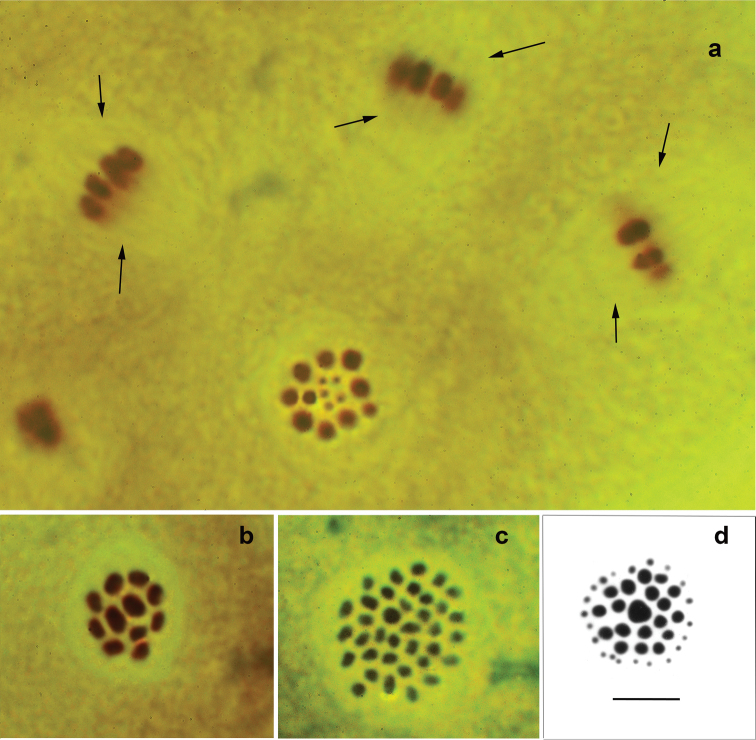
Intact male meiotic metaphase I plates in Ithomiini (**a–c**) and Polyommatini (**d**) butterflies **a***Forbestraolivenciaolivencia* (Bates, 1862), n=15 (10 macrobivalents + 5 microbivalents); three metaphase plates on the top are from the side (=equatorial) view; the plate on the bottom is from pole view; meiotic spindle is indicated by arrows **b***Oleriagunillaserdolis* (Haensch, 1909), n=11 **c***Godyrisdircenna* (C. Felder et R. Felder, 1865), n=36 **d**Polyommatus (Agrodiaetus) dagestanicus (Forster, 1960), n=40 (19 macrobivalents + 21 microbivalents) (from Lukhtanov and Dantchenko 2002). Scale bar: 10 μ in all figures.

The meiotic karyotype of *Forbestraolivenciaolivencia* was found to include 9 large and 1 medium elements (interpreted as 10 macrobivalents) and 5 very small elements (interpreted as 5 microbivalents) (Fig. 2a). Thus, the karyotype is similar (but not exactly identical) to the previously studied karyotypes of *F.olivencia* and *F.proceris* for which a low basic haploid number (nine) and a variable amount (from one to eight) additional minute chromosome elements were reported (Brown et al. 2004).

In all studied metaphase plates the same picture was observed: the species showed the distinct disk-like structure of the metaphase I plates, having all the bivalents inside the meiotic spindle. The structure of the intact metaphase I plates was simple and stable. Large bivalents (i.e. pairs of macrochromosomes) formed a more or less regular peripheral circle, whereas the minute bivalents (i.e. pairs of microchromosomes) occupied the center of this circle. Thus, *F.olivencia* has typical “reptilian” type (the terminology of White 1973) of the spatial organization of bivalents.

The meiotic karyotype of *Oleriagunillaserdolis* was found to include 11 bivalents (Fig. 2b) confirming results of the previous cytogenetic analysis of this species (Brown et al. 2004). Two bivalents were significantly larger than the other nine ones. These two larger bivalents occupied the position in the center of metaphase plate in accordance with observation on other Lepidoptera (Lukhtanov and Dantchenko 2002). Thus, *O.gunillaserdolis* has the typical “lepidopteran” type of the spatial organization of bivalents.

The meiotic karyotype of *Godyrisdircenna* was found to include 36 bivalents (Fig. 2c) confirming results of the previous cytogenetic analysis of this species (Brown et al. 2004). The bivalents had different sizes and shapes. One bivalent was slightly larger than the rest ones and had a tendency to be located in the center of metaphase plate in accordance with observation on other Lepidoptera (Lukhtanov and Dantchenko 2002). Thus, *Godyrisdircenna* has the “lepidopteran” type of the spatial organization of bivalents.

The spatial arrangement of the large and small bivalents in *Forbestraolivencia* is fundamentally different from the structure found in this and in previous studies in other butterflies, e.g. in Polyommatus (Agrodiaetus) dagestanicus (Forster, 1960) (Fig. 2d). In the latter species the bivalents show a regular concentric arrangement with the largest bivalent situated in the central part of the rounded metaphase plate. The medium bivalents formed two internal rings and the microelements formed an external, peripheral ring of the metaphase plate.

Previously we hypothesized that the lepidopteran type of the metaphase plate organization in butterflies can be explained by holocentric nature of their chromosomes, which are characterized by kinetic activity distributed along almost the entire chromosome length (Lukhtanov et al. 2018). We suggested that during congregation at the prometaphase stage there was a centripetal movement of bivalents made by a pulling force directed to the centre of the metaphase plate transverse to spindle. The magnitude of this force may be depending on the quantity of microtubules contacted to the chromosome and, correspondingly, on the kinetochore size. Therefore, large bivalents having large kinetochores were situated in the central part of metaphase plate (Lukhtanov and Dantchenko 2002). However, the unusual organization of metaphase plate in *F.olivencia* demonstrates that the suggested explanation is not universal and not necessarily true. Recently, McClure et al. (2017) hypothesized that some Ithomiini butterflies had an atypical holocentric chromosomes, and each anaphasic chromosome seemed to be driven by a single microtubule, and not by multiple ones. This hypothesis, if it is true, can explain the unusual structure of metaphase plate in *Forbestraolivencia*, but first this hypothesis itself must be tested.
